# Life as a Chronically Ill PhD Student

**DOI:** 10.1021/acscentsci.3c00464

**Published:** 2023-05-11

**Authors:** Libby Brown

*Libby Brown, a final year PhD student at the University of Cambridge, shares her experience of completing a PhD with a chronic illness and describes how flexible working, open communication, and university support can foster diversity within academia.*

Twenty percent of PhD students suffer from a chronic mental or physical health condition. Yet, when searching for resources and support for those affected, there is something of a void. For most of my life, I have been described as a medical mystery. I suffer from fatigue and chronic pain and am regularly admitted to hospital for the treatment of kidney, liver, lung...(the list is endless) infections. These hospitalizations generally follow periods of high stress or high workload, both of which are to be expected during a PhD.

Here, I discuss the challenges I have faced as a chronically ill PhD student. A PhD is no mean feat on its own, but combined with illness, it becomes 10 times tougher. However, adaptations can be made to make the experience less challenging and more enjoyable.

## Financial Considerations

Many of the challenges faced by chronically ill PhD students stem from a lack of employee status. Sick pay, reduced hours, and phased return to work are not luxuries granted to all PhD students. While my funding body has a sick leave policy that entitles each student to 13 weeks of leave per academic year, gathering the information and filling in the forms required to receive an extension can be difficult and time-consuming. Furthermore, moving even temporarily to part-time status can trigger a stipend reduction. Many PhD students are not in a financial position to consider this option as, in Cambridge, for example, the full-time stipend only just covers the cost of living.

In my case, my PhD supervisor (Professor Gonçalo Bernardes, who suffers from ankylosing spondylitis, an autoimmune disease) was willing to cover the additional costs associated with moving to part-time status. By reducing my work hours, I was able to manage my fatigue, work more efficiently, and improve my general well-being (so much so that I have now returned to full-time work!). While an informal discussion of my work hours worked for me, it may not be a viable option for all PhD students. It relies on the availability of additional funding and your supervisor’s willingness to adapt expectations.

## Communication

There is still a significant stigma associated with chronic and invisible health conditions. And, it is understandable that students may not wish to disclose their health condition out of fear of disbelief, pity, or being treated differently. There is no obligation to inform your university of a pre-existing health condition and access to other support networks should still be available without doing so.

Having said this, I initiated open communication with my supervisor and university postgraduate support staff from the start, finding them to be a great source of advice and understanding. By discussing my illness, I have been able to establish a working relationship with my supervisor that considers the extra challenges I face and adapt my working conditions to keep me as healthy and happy as possible.

## Work flexibility within an academic institution

Despite the obstacles I have already discussed, academia can provide a great working environment for those coping with chronic illness. PhD’s generally offer high levels of flexibility and freedom. For example, when my concentration levels are high, I can read papers and write up experiments. When my focus is low, I work on simple lab tasks and reply to emails. This situation is only possible as I work on a solo project, and, as such, colleagues are not reliant on me being in a certain place at a certain time. Most PhD students will have access to disability advisors, counsellors, and specialist equipment (if needed) through their university. And, if a long-term health condition or disability makes certain aspects of postgraduate study more expensive, additional funding can be applied for.

## To summarize...

Completing a PhD program as a chronically ill student is hard but possible with support from your supervisor, university, and colleagues. What’s more, the positive qualities learned from managing a chronic illness—problem-solving, note-taking, resilience, independence, and time management—make those affected well-equipped for a career in research. What you have read today is my personal experience. Undoubtedly, others at different universities with different health conditions, supervisors, and support will have very different experiences. Researchers with chronic health conditions and disabilities are underrepresented at the PhD, postdoc level, and beyond, with significantly higher dropout levels recorded for those registered with a physical or mental health condition. This situation suggests that issues such as accessibility, stigmatization, and lack of work flexibility are still prominent within the academic community. University education is an important catalyst for improving social mobility and employability among underrepresented groups, including those with chronic illnesses. Therefore, academic institutions must continue to promote diversity, ensuring that good quality working conditions and support are available for chronically ill students and members of staff.

